# Preliminary Findings from Three Models of Motivational Interviewing Training in Jamaica

**DOI:** 10.1089/heq.2020.0034

**Published:** 2020-10-07

**Authors:** Henna Budhwani, Sylvie Naar

**Affiliations:** ^1^ University of Alabama at Birmingham (UAB), School of Public Health, Department of Health Care Organization and Policy, Birmingham, Alabama, USA.; ^2^ Florida State University (FSU), College of Medicine, Center for Translational Behavioral Science, Tallahassee, Florida, USA.

**Keywords:** HIV, Jamaica, Implementation Science, motivational interviewing, youth, capacity building

## Abstract

**Introduction:** We assessed satisfaction, fidelity, retention, and implementation considerations across three models of motivational interviewing training in Jamaica to identify a promising model for resource-poor settings.

**Methods:** We conducted *t*-tests to assess differences in fidelity and examined qualitative data for barriers and facilitators (*n*=52).

**Results:** Only 50−75% of all models' trainees completed coaching. Model 1 trainees' mean fidelity was 2.83/4.00 compared with Model 3 trainees' at 2.94/4.00 (*t*=−0.710, confidence interval=−0.427 to 0.207, *p*=0.483). Key barriers to completion and fidelity were lack of funding and time.

**Conclusion:** We found support for continuing workshop-only trainings; we did not find that higher contact hours produced improved trainee fidelity.

## Introduction

Motivational interviewing (MI)—when embedded in clinical settings—improves health outcomes across the HIV continuum of care.^1–6^ As such, MI has been included as part of the recommended standard of care for youth living with HIV (YLWH) in the United States.^2–6^ Training providers in MI and supporting them to attain fidelity can be time intensive and costly,^7–10^ and characteristics of organizational settings can act as barriers to implementing MI with fidelity.^11^ Barriers may be exacerbated in resource-constrained settings, but we know that when interventionists are able to culturally tailor models for local contexts, then levels of training satisfaction are higher, and trainees are likely to be more adherent to protocols, which then may lead to higher fidelity outcomes, ultimately leading to improved health equity.^12^

Jamaica is a resource-constrained nation struggling with a concentrated HIV epidemic among youth. Jamaica has the second highest HIV prevalence in the Caribbean with a staggering 32% in young men who have sex with men.^13–16^ AIDS is a leading cause of death in Jamaican youth; only 21% of Jamaican YLWH are on antiretroviral therapy, and a mere 5% achieve viral suppression.^17–20^ Experts partially attribute these failures to stigmatizing attitudes in health care settings toward YLWH.^21–24^ Past interventions have failed in changing behaviors, in part, because youth are unwilling to disclose their risk behaviors due to stigma emerging in patient–provider communications.^11^ If a model of MI delivery can be refined to promote fidelity, such a model has the potential to improve the in-clinic experiences of Jamaican YLWH.

Considering structural barriers alongside the potential positive impact of MI on Jamaica's HIV continuum of care, our team conducted a pilot study with the objectives of (1) reporting on trainee fidelity and retention, (2) assessing for a significant difference in fidelity attained by trainees who participated in the lowest contact hour model compared with the highest contact hour model, and (3) characterize barriers and facilitators to MI implementation in Jamaica.^13–16^

## Methods

### Data and sample

Participants (*n*=52) were HIV providers and prevention and outreach workers in Jamaica who had at least 4 h of HIV-related client contact per week (2015–2017). Most providers had some high school level education, and a smaller proportion had college-level educational attainment. All participants were adults (aged >18 years). Participants were identified through consultation with the Jamaica Ministry of Health and the Caribbean Vulnerable Communities Coalition. Randomization was not employed. Trainees only participated in one model. Study participants were not provided any financial incentive.

### Ethics

Approval was provided by the Florida State University Institutional Review Board (#00000287).

### Framework

We applied the Exploration Preparation Implementation Sustainment (EPIS) framework^25^ to inform the design of this study and used the framework to classify the inner and outer contexts affecting implementation.

### Models

Model 1 included a 2-day face-to-face workshop (12 h) conducted by American, Motivational Interviewing Network of Trainers (MINTs) followed by six individual MI coaching sessions (1 h each) conducted through Skype or phone call. Model 1 (*n*=20) included a maximum of 18 hours of contact. Model 2 reduced the number of coaching sessions from six to four and added two face-to-face booster workshops (6 hours of contact per booster). Model 2 (*n*=20) included 28 hours of contact. Model 3 included a 3-day workshop (18 h), two face-to-face booster, two one-on-one coaching sessions, and was conducted by Jamaican facilitators. Model 3 (*n*=12) included 32 hours of contact. See Table 1 for features of each model (*n*=52).

**Table 1.** Features of Three Models of Motivational Interviewing Training in Jamaica

	Model 1	Model 2	Model 3
No. of workshop attendees	20	20	12
American MINTs	Yes	Yes	No
Jamaican MINT	No	No	Yes
Jamaica facilitators	No	Yes	Yes
Nonprovider stakeholders	Yes	No	No
Providers	Yes	Yes	Yes
Workshop length	12 h	12 h	18 h
1-on-1 coaching	Yes	Yes	Yes
No. of coaching sessions	6	4	2
Boosters workshops	No	Yes	Yes

MINT, Motivational Interviewing Network of Trainer.

### Measures: Workshop satisfaction

Written pre- and postworkshop satisfaction assessments (quantitative and qualitative) were conducted on paper. Satisfaction was assessed by questions relating to whether topics were relevant, training was useful, trainers were knowledgeable, and whether trainees felt more confident in the delivery MI. Participants were also asked to provide written feedback postworkshop and postbooster (when applicable).

### Measures: Fidelity

MI fidelity was measured through the conduct and coding of standard patient roleplays using the MI coach rating scale (MI-CRS).^12^ Trainees participated in audiorecorded standard patient roleplays, which were scored after the session. The MI-CRS was developed using item response theory and includes 12 items assessing competence on a 4-point Likert scale ranging from poor to excellent.^12^ Competency thresholds are beginner (<2.0), novice (2.0–2.6), intermediate (2.7–3.3), and advanced (>3.3).^15^

### Analyses

We conducted an independent samples *t*-test using SPSS 25 to assess whether there were statistical differences in mean fidelity between the highest and lowest contact hour models. To classify implementation barriers and facilitators, we leveraged elements of the thematic analysis approach. Owing to the limited nature of our data, we only identified themes and then assigned those themes as being either a barrier or facilitator, thereafter as being embedded within the inner or outer context, as defined by the EPIS framework.^25^

## Results

### Satisfaction

Overall workshop satisfaction was high across all models. Satisfaction ranged across measures and models from 75% to 100% were satisfied.

### Model 1: Workshop+coaching+American

The following statement aptly summarized participant sentiments after the initial workshops:

“Initially, I thought it was a joke to attend the workshop. But coming and reflecting on the skillset, it is needed in my job and will enhance my skill and transform my approach in doing things… I appreciate the opportunity. It was not boring and was creative.”

Although satisfaction was high, coaching completion was low. Sixty percent of Model 1's participants entered coaching; 50% completed three or more coaching sessions (*n*=10). The mean number of coaching sessions was 3.18 (standard deviation [SD]=2.04) sessions. The mean participant fidelity score was 2.83 (SD=0.34, intermediate competency). See Table 2 for results from Model 1.

**Table 2.** Blinded Motivational Interviewing Fidelity Results from Model 1 (*n*=20)

Trainee	No. of coaching sessions	Most recent fidelity score	Highest fidelity score	Average fidelity score
A	0	0.00	0.00	0.00
B	0	0.00	0.00	0.00
C	0	0.00	0.00	0.00
D	1	3.33	3.33	3.33
E	1	2.92	2.92	2.92
F	1	2.67	2.67	2.67
G	1	2.25	2.25	2.25
H	1	3.08	3.08	3.08
I	1	3.08	3.08	3.08
J	1	2.67	2.67	2.67
K	3	3.00	3.08	2.94
L	3	2.50	2.83	2.58
M	4	3.08	3.08	2.23
N	4	2.67	2.67	2.50
O	4	2.75	2.75	2.56
P	5	2.50	2.83	2.68
Q	6	3.58	3.58	3.22
R	6	3.92	3.92	3.38
S	6	3.50	3.50	2.97
T	6	3.67	3.67	2.99

### Model 2: Workshop+coaching+boosters+American

Barriers were uncovered and addressed during the adaptation of Model 1 to Model 2, including the inability to record live interactions with patients (shifted to using standard patient roleplays), difficultly using the scheduling software (used e-mail to schedule outside of the system), and unstable Internet (provided phone cards for international calling). Considering cultural contexts, we modified the postworkshop process to reduce the coaching sessions from six to four while adding two face-to-face boosters. Feedback solicited during the booster sessions was positive:

“[MI] allows me confidence and control in unknown client situations; provide clarity in connecting client care, client needs and goal aimed at HIV treatment.”

Even with this positive feedback, in Model 2 (*n*=20) coaching attrition stayed the same with ∼50% of trainees completing at least three sessions. Six workshop attendees (30%) did not complete any coaching. Fourteen trainees (70%) scheduled a coaching session but then did not show up. The mean number of coaching sessions of those who completed at least one coaching session was 3.29 (SD=1.10) sessions. Attendance in the face-to-face booster sessions was strong with about two-thirds of trainees attending these sessions.

### Model 3: Workshop+coaching+boosters+Jamaican

Model 3 was conducted by Jamaican trainers (*n*=12); the workshop was extended from 2 to 3 days. Two face-to-face boosters were retained from Model 2, plus two one-on-one coaching sessions. The mean of each participant's average fidelity score was 2.94 (SD=0.54), indicating intermediate competency. A trainee comment from Model 3 was that:

“Everyone appreciated the presentation style as it allowed for participation; it was experiential. I had no need to worry. Appreciated being stretched out of my comfort zone… I believe it will be useful. I spoke yesterday with my husband about MI and gave the example of when the client says, ‘I don't know.’ It was an epiphany for me…”

### Comparing Model 1 with Model 3 outcomes

Model 1 included the fewest contact hours and Model 3 included the most contact hours. About 50% of Model 1's trainees completed their coaching; comparatively, about 75% of Model 3's trainees completed their coaching. Trainees from both attained, on average, intermediate level of competency. The average fidelity score for Model 1 trainees was 2.83 compared with 2.94 for Model 3 trainees (*t*=−0.710, confidence interval=−0.427 to 0.207, *p*=0.483), indicating no statistically significant difference.

### Inner and outer contexts

Using the EPIS framework,^25^ we categorized the inner and outer contexts influencing MI delivery in Jamaica for use by public health scholars and practitioners who conduct intervention research or seek to build capacity by delivering MI Table 3.

**Table 3.** Inner and Outer Contexts Potentially Influencing Intervention Fidelity

	Barriers	Facilitators
Inner contexts	Funding to support trainee time Insufficient broadband access in some agencies Inability to access phones or computers for ongoing coaching	Organization leadership support Culture of quality improvement Sufficient staffing to delivery of HIV care to youth
Outer contexts	Sociopolitical environment that stigmatizes YLWH, so that trainees may not be invested in the intervention Shifting funding priorities Incentive expectations set by other American and European capacity building groups	Ongoing coaching MI fidelity monitoring Use to expert MI trainers to deliver content and coaching.

MI, motivational interviewing; YLWH, youth living with HIV.

## Discussion

We found some evidence to support the value of continuing to deliver workshop-only trainings (current standard), but we did not find evidence to support generally held belief that higher face-to-face contact models produce higher fidelity.

### Limitations

Detailed participant demographic data were not collected, so we were unable to examine outcomes by age, gender, etc. Contamination could have led to improved outcomes in Models 2 and 3. Self-selection bias may affect study outcomes; participants who decided to enroll in this study may have held different personal characteristics than those who opted out of participating.

### Future research

Since interventions shown to be effective in the United States may not be translatable without substantial adaptation for local contexts, future research examining the implementation of behavioral interventions in resource-constrained settings continues to be warranted.

## Conclusion

Delivering MI with fidelity in settings affected by concentrated HIV epidemics may be accomplished if structural limitations are considered, but also requires attention to implementation contexts, if health equity is to be attained.

## Abbreviations Used

EPIS: Exploration Preparation Implementation Sustainment
MI: motivational interviewing
MI-CRS: motivational interviewing coach rating scale
MINT: Motivational Interviewing Network of Trainer
SD: standard deviation
YLWH: youth living with HIV
